# Lung immune prognostic index as a prognostic factor in patients with small cell lung cancer

**DOI:** 10.1111/1759-7714.13432

**Published:** 2020-04-14

**Authors:** Kei Sonehara, Kazunari Tateishi, Masamichi Komatsu, Hiroshi Yamamoto, Masayuki Hanaoka

**Affiliations:** ^1^ First Department of Internal Medicine Shinshu University School of Medicine Matsumoto City Japan

**Keywords:** Derived neutrophil‐to‐lymphocyte ratio, lung immune prognostic index, prognostic factor, serum lactate dehydrogenase, small cell lung cancer

## Abstract

**Background:**

The lung immune prognostic index (LIPI) is a marker that combines the derived neutrophil‐to‐lymphocyte ratio (dNLR) and serum lactate dehydrogenase (LDH) level and is a recently reported prognostic factor of immune checkpoint inhibitor therapy for non‐small cell lung cancer (NSCLC). However, there are no reports regarding the prognostic value of LIPI in small cell lung cancer (SCLC).

**Methods:**

We retrospectively enrolled 171 patients diagnosed with SCLC and treated at Shinshu University School of Medicine between January 2003 and November 2019. Progression‐free survival (PFS) and overall survival (OS) were compared according to LIPI, and we investigated whether LIPI could be a prognostic factor in SCLC using the Kaplan‐Meier method and univariate and multivariate Cox models.

**Results:**

The median OS of the LIPI 0 group was significantly longer than that of the LIPI 1 plus 2 group (21.0 vs. 11.6 months, *P* < 0.001). The multivariate analysis associated with OS indicated that LIPI 1 plus 2 was an independent unfavorable prognostic factor in addition to poor performance status (2–3), old age (≥ 75 years) and stage (extensive disease [ED]). However, PFS of the LIPI 0 group was not significantly different from that of the LIPI 1 plus 2 group. In ED‐SCLC patients, the median PFS and OS of the LIPI 0 group were significantly longer than those of the LIPI 2 group (6.6 vs. 4.0 months, *P* = 0.006 and 17.1 vs. 5.9 months, *P* < 0.001, respectively).

**Conclusions:**

We confirmed the prognostic value of LIPI in SCLC, especially ED‐SCLC.

**Key points:**

- Significant findings of the study: The present study is the first to demonstrate that pretreatment lung immune prognostic index is an independent prognostic factor associated with overall survival for small cell lung cancer.
- What this study adds: The utility of the lung immune prognostic index as a prognostic factor for small cell lung cancer.

## Introduction

Small cell lung cancer (SCLC) is a solid tumor characterized by rapid progression and early development of metastases that accounts for approximately 13%–15% of all cases of lung cancer; one‐third of these cases are classified as limited disease (LD), and two‐thirds as extensive disease (ED).^1^, ^2^ Treatment approaches are used to determine whether LD or ED and staging are the most important prognostic factors associated with overall survival (OS).^3^ Previous reports have suggested that patient characteristics such as performance status (PS), age, smoking status and staging are prognostic factors in patients with SCLC.^3^, ^4^ Systemic immune and inflammatory status in the body are critical in cancer prognosis. Many recent reports have suggested that various markers of systemic inflammation, such as the neutrophil‐to‐lymphocyte ratio (NLR), platelet/lymphocyte ratio (PLR), systemic immune‐inflammation index (SII), and modified Glasgow prognostic score (mGPS), would be useful as prognostic factors in SCLC patients.^4^, ^5^, ^6^, ^7^, ^8^ It is also important that these biomarkers may be available in all institutions and are cost‐effective. The lung immune prognostic index (LIPI) is a marker combining the derived neutrophil‐to‐lymphocyte ratio (dNLR) and serum lactate dehydrogenase (LDH) level and has recently been reported as a prognostic factor of immune checkpoint inhibitors for non‐small cell lung cancer (NSCLC).^9^, ^10^ LIPI categorizes patients in three groups as dNLR and LDH are routinely available markers in daily clinical practice. In brief, patients with dNLR greater than 3 and LDH higher than the upper limit of normal (ULN) are defined as “Poor (LIPI 2)”, patients with dNLR greater than 3 and LDH lower than ULN or dNLR less than 3 and LDH higher than ULN are defined as “Intermediate (LIPI 1)”, and patients with dNLR less than 3 and LDH lower than ULN are defined as “Good (LIPI 0)”.^9^ dNLR and LDH have previously been shown to be useful prognostic factors in many studies.^11^, ^12^, ^13^ Thus, LIPI with a clear cutoff value is expected to be a useful marker. A previous retrospective report suggested that LIPI is a useful marker for chemotherapy and epidermal growth factor receptor tyrosine kinase inhibitor for NSCLC.^14^ However, there have been no reports describing the prognostic value of LIPI in SCLC. Therefore, in the present study, we investigated the clinical significance of LIPI as a useful prognostic factor focusing on progression‐free survival (PFS) and OS in SCLC. We also examined whether the usefulness of LIPI depends on the stage (LD or ED).

## Methods

The present study was retrospectively conducted and approved by the institutional review board of Shinshu University School of Medicine (approval number 4673). All data were conducted in accordance with the principles of the Declaration of Helsinki. Between January 2003 and November 2019, patients with SCLC diagnosed and treated at Shinshu University Hospital were enrolled. Using electronic medical records we searched the information on each patient. Individual patient information was protected and has not been shown.

According to the World Health Organization classification, version 7, a histological diagnosis was made. SCLC was classified according to the eighth edition of the TNM classification. The Eastern Cooperative Oncology Group PS was evaluated at the time of diagnosis, and the best objective response to treatment was evaluated using the Response Evaluation Criteria in Solid Tumors, version 1.1. Patient information, such as age (< 75 years vs. ≥ 75 years), PS (0–1 vs. 2–3), smoking history (never vs. current plus former), interstitial pneumonia (without vs. with), stage (LD vs. ED), and LIPI (0 vs. 1 plus 2), were collected for the analysis. The cutoff value of LDH was determined based on the ULN. LD was defined by lesions limited to one hemithorax, regional mediastinal lymph nodes, and ipsilateral supraclavicular lymph nodes and can be encompassed within a tolerable radiation field; ED was defined as other cases not included in LD.^15^ The objective response rate (ORR) was defined as the complete response (CR) rate or partial response (PR) rate. PFS and OS were defined as the time from the initial treatment date to the date of progressive disease (PD) and the interval from the date of diagnosis, or to the date of death or the last follow‐up visit, respectively. PFS and OS were compared between the LIPI 0 group and the LIPI 1 plus 2 group. With regard to ED‐SCLC, LIPI was used to divide patients into three groups to evaluate the ORR and one‐year survival rate, as well as how chemotherapy was administered according to the LIPI value.

### Statistical analysis

The PFS and OS analyses of all SCLC patients and those of LD‐ or ED‐SCLC patients were evaluated using the Kaplan‐Meier method. Significance tests for PFS and OS were compared using the log‐rank test. Univariate and multivariate analyses using the Cox proportional hazard model were performed to determine independent prognostic factors. The date of last follow‐up in the present study was 31 January 2020. Comparisons between the groups were analyzed using Fisher's exact test, and a *P*‐value of <0.05 indicated statistical significance. Statistical analysis was performed using IBM SPSS Statistics, version 26.

## Results

### Patient characteristics

A total of 171 SCLC patients were included in the present study between January 2003 and November 2019. There were 66 and 105 patients classified as having LD‐SCLC and ED‐SCLC, respectively. The clinical characteristics of the patients are summarized in Table 1. The median age was 70 years (range: 43–87 years), and there were 146 men (85.4%) and 25 women (14.6%) in the study population. At the time of pretreatment, 45 patients (26.3%) had PS 0, 93 patients (54.4%) had PS 1, 20 patients (11.7%) had PS 2, and 13 patients (7.6%) had PS 3. A total of 159 patients (93.0%) were smokers, and 12 (7.0%) were never smokers. A total of 31 patients (18.1%) had been diagnosed with interstitial pneumonia. According to the eighth edition of the TNM classification, 18 (10.5%), 54 (31.6%), and 99 (57.9%) patients were classified as stage I–II, III, and IV, respectively. A total of 76 patients (44.4%) had LDH < 223 U/L, and 95 (55.6%) had LDH ≥223 U/L. The median dNLR was 2.1 (range: 0.7–15.6). According to the LIPI value, 64 (37.4%), 79 (46.2%), and 28 (16.4%) patients were classified into the LIPI 0, LIPI 1, and LIPI 2 groups, respectively. With regard to first‐line treatment, chemoradiotherapy, chemotherapy, radiotherapy, surgical operation, and palliative care were administered to 28 (16.4%), 135 (78.9%), two (1.2%), three (1.8%), and three (1.8%) patients, respectively.

**Table 1:** Patient characteristics

Category	All SCLC patients, N (%)	LD‐SCLC patients, N (%)	ED‐SCLC patients, N (%)
Patients (N)	171	66	105
Median age (range), years	70 (43‐87)	69 (51‐87)	71 (43‐86)
Gender, male/female	146 (85.4)/25 (14.6)	55 (83.3)/11 (16.7)	91 (86.7)/14 (13.3)
ECOG performance status			
0	45 (26.3)	22 (33.3)	23 (21.9)
1	93 (54.4)	39 (59.1)	54 (51.4)
2	20 (11.7)	3 (4.5)	17 (16.2)
3	13 (7.6)	2 (3.0)	11 (10.5)
Smoking history current plus former/never	159 (93.0) /12 (7.0)	59 (89.4)/7 (10.6)	100 (95.2)/5 (4.8)
Interstitial pneumonia with/without	31 (18.1) /140 (81.9)	15 (22.7)/51 (77.3)	16 (15.2)/89 (84.8)
Staging			
I–II	18 (10.5)	18 (27.3)	0 (0.0)
IIIA/IIIB/IIIC	28 (16.4)/16 (9.4)/10 (5.8)	28 (42.4)/16 (24.2)/ 4 (6.1)	—/—/6 (5.7)
IVA/IVB	32 (18.7)/67 (39.2)	—/—	32 (30.5)/67 (63.8)
Laboratory data			
Alb (g/dL) <3.5 /≥3.5	40 (23.4) /131 (76.6)	8 (12.1)/58 (87.9)	32 (30.5)/73 (69.5)
CRP (mg/dL) <1.0 /≥1.0	117 (68.4) /54 (31.6)	54 (81.8)/12 (18.2)	63 (60.0) 42 (40.0)
LDH (U/L) <223/≥223	76 (44.4) /95 (55.6)	37 (56.1)/29 (43.9)	39 (37.1)/66 (62.9)
dNLR (range)	2.1 (0.7‐15.6)	1.8 (0.8‐5.1)	2.4 (0.7‐15.6)
LIPI, 0/1/2	64 (37.4)/79 (46.2)/28 (16.4)	34 (51.5)/27 (40.9)/5 (7.6)	30 (28.6)/52 (49.5)/23 (21.9)
First‐line treatment			
Chemoradiotherapy	28 (16.4)	28 (42.4)	0 (0.0)
Chemotherapy	135 (78.9)	33 (50.0)	102 (97.1)
Radiotherapy	2 (1.2)	2 (3.0)	0 (0.0)
Surgical operation	3 (1.8)	3 (4.5)	0 (0.0)
Best supportive care	3 (1.8)	0 (0.0)	3 (2.9)

dNLR, derived neutrophils/(leukocytes minus neutrophils) ratio; ECOG, Eastern Cooperative Oncology Group; ED, extensive disease; LD, limited disease; LDH, lactate dehydrogenase; LIPI, lung immune prognostic index; SCLC, small cell lung cancer.

### Prognostic factors associated with PFS for SCLC patients

The results of univariate and multivariate analyses of factors associated with PFS are summarized in Table 2. Multivariate analysis revealed that PS 0–1 (HR 1.52, 95% CI: 1.20–1.93, *P* = 0.001), never‐smoker status (HR 2.46, 95% CI: 1.13–5.36, *P* = 0.024), absence of interstitial pneumonia (HR 1.72, 95% CI: 1.08–2.72, *P* = 0.022), and LD (HR 2.49, 95% CI: 1.66–3.74, *P* < 0.001) were independent favorable prognostic factors. Regarding LIPI, there was a significant difference between the LIPI 0 group and the LIPI 1 plus 2 group in the univariate analysis (0 vs. 1 plus 2: HR 1.53, 95% CI: 1.07–2.20, *P* = 0.020), but there was no significant difference in the multivariate analysis (0 vs. 1 plus 2: HR 1.23, 95% CI: 0.83–1.81, *P* = 0.296).

**Table 2:** Univariate and multivariate Cox hazard analysis of potential factors associated with progression‐free survival

	Univariate				Multivariate		
Category	PFS (months)	HR	95% CI	*P*‐value	HR	95% CI	*P*‐value
Age, years							
<75 vs. ≥75	6.7 vs. 6.3	1.27	0.85–1.89	0.246			
ECOG performance status							
0–1 vs. 2–3	7.2 vs. 4.1	1.61	1.29–2.00	<0.001	1.52	1.20–1.93	0.001
Smoking history never vs. current plus former	8.7 vs. 6.5	2.32	1.08–5.00	0.026	2.46	1.13–5.36	0.024
Interstitial pneumonia without vs. with	6.7 vs. 6.5	1.58	1.01–2.46	0.047	1.72	1.08–2.72	0.022
Stage							
LD vs. ED	8.9 vs. 5.4	2.88	1.94–4.27	<0.001	2.49	1.66–3.74	<0.001
LIPI							
0 vs. 1‐2	7.6 vs 5.8	1.53	1.07–2.20	0.020	1.23	0.83–1.81	0.296

ECOG, Eastern Cooperative Oncology Group; ED, extensive disease; LD, limited disease; LIPI, lung immune prognostic index; PFS, progression‐free survival.

### Prognostic factors associated with OS for SCLC patients

The results of univariate and multivariate analyses of factors associated with OS are summarized in Table 3. Multivariate analysis revealed that age < 75 years (HR 1.67, 95% CI: 1.14–2.42, *P* = 0.008), PS 0–1 (HR 1.53, 95% CI: 1.22–1.91, *P* < 0.001), LD (HR 2.18, 95% CI: 1.47–3.23, *P* < 0.001), and LIPI 0 (HR 1.63, 95% CI: 1.11–2.40, *P* = 0.013) were independent favorable prognostic factors.

**Table 3:** Univariate and multivariate Cox hazard analysis of potential factors associated with overall survival

	Univariate				Multivariate		
Category	OS (months)	HR	95% CI	*P*‐value	HR	95% CI	*P*‐value
Age, years							
<75 vs. ≥75	16.1 vs. 12.9	1.56	1.08–2.26	0.017	1.67	1.14–2.42	0.008
ECOG performance status							
0–1 vs. 2–3	16.6 vs. 6.7	1.79	1.46–2.21	<0.001	1.53	1.22–1.91	<0.001
Smoking history never vs. current plus former	14.8 vs. 15.2	1.32	0.67–2.60	0.423			
Interstitial pneumonia without vs. with	16.0 vs. 14.5	1.37	0.87–2.16	0.180			
Stage							
LD vs. ED	23.8 vs. 11.6	2.62	1.80–3.88	<0.001	2.18	1.47–3.23	<0.001
LIPI							
0 vs. 1–2	21.0 vs. 11.6	2.07	1.44–2.96	<0.001	1.63	1.11–2.40	0.013

ECOG, Eastern Cooperative Oncology Group; ED, extensive disease; LD, limited disease; LIPI, lung immune prognostic index; OS, overall survival.

### PFS and OS for all‐SCLC, LD‐SCLC and ED‐SCLC patients

**Figure 1:**
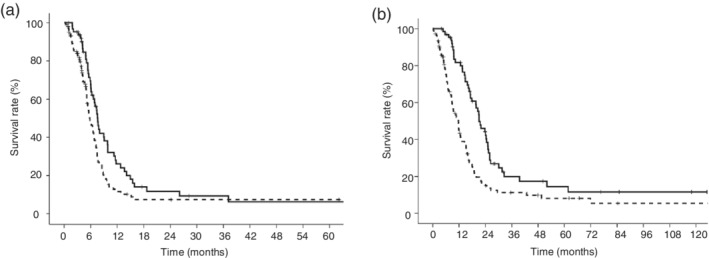
Kaplan‐Meier curves according to the lung immune prognostic index (LIPI) in small cell lung cancer (SCLC) patients. (**a**) The median progression‐free survival (PFS) of the LIPI 0 group was significantly longer than that of the LIPI 1 plus 2 group (7.6 months vs. 5.6 months, respectively, *P* = 0.020) (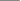 ) LIPI 0 group, (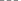 ) LIPI 1 plus 2 group. (**b**) The median overall survival (OS) of the LIPI 0 group was significantly longer than that of the LIPI 1 plus 2 group (21.0 months vs. 11.6 months, respectively, *P* < 0.001) (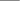 ) LIPI 0 group, (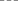 ) LIPI 1 plus 2 group.

The PFS and OS of SCLC patients are shown in Figure 1. The PFS of the LIPI 0 group and LIPI 1 plus 2 group was 7.6 months (95% CI: 6.7–8.5 months) and 5.8 months (95% CI: 4.8–6.9 months), respectively. The OS of the LIPI 0 group and LIPI 1 plus 2 group was 21.0 months (95% CI: 17.2–24.9 months) and 11.6 months (95% CI: 9.2–14.0 months), respectively. The PFS and OS of the LIPI 0 group were significantly longer than those of the LIPI 1 plus 2 group (*P* = 0.020 and *P* < 0.001, respectively).

**Figure 2:**
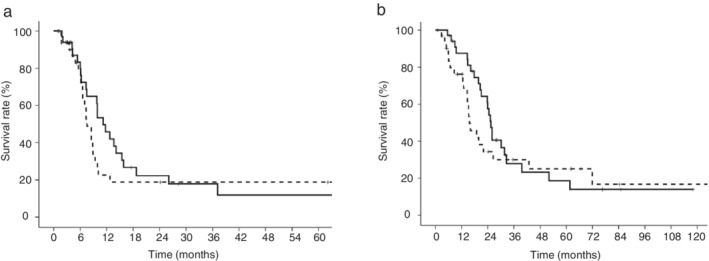
Kaplan‐Meier curves according to the lung immune prognostic index (LIPI) in LD‐SCLC patients. (**a**) The median progression‐free survival (PFS) of the LIPI 0 group was not significantly different from that of the LIPI 1 plus 2 group (11.2 months vs. 7.6 months, respectively, *P* = 0.397) (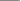 ) LIPI 0 group, (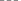 ) LIPI 1 plus 2 group. (**b**) The median overall survival (OS) of the LIPI 0 group was not significantly different from that of the LIPI 1 plus 2 group (25.5 months vs. 15.6 months, respectively, *P* = 0.383) (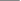 ) LIPI 0 group, (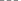 ) LIPI 1 plus 2 group.

The PFS and OS in LD‐SCLC patients are shown in Figure 2. The PFS of the LIPI 0 group and LIPI 1 plus 2 group was 11.2 months (95% CI: 8.3–14.1 months) and 7.6 months (95% CI: 6.1–9.1 months), respectively. The OS of the LIPI 0 group and LIPI 1 plus 2 group was 25.5 months (95% CI: 23.4–27.5 months) and 15.6 months (95% CI: 11.1–20.1 months), respectively. The PFS and OS of the LIPI 0 group were not significantly different from those of the LIPI 1 plus 2 group (*P* = 0.397 and *P* = 0.383, respectively).

**Figure 3:**
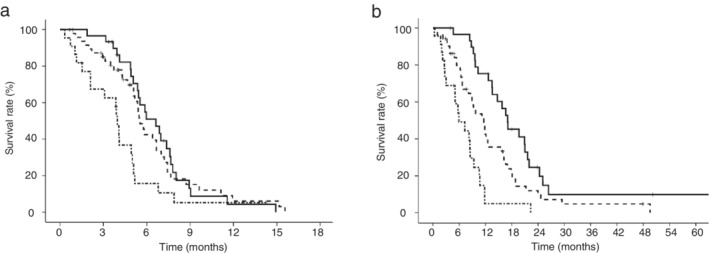
Kaplan‐Meier curves according to the lung immune prognostic index (LIPI) in ED‐SCLC patients. (**a**) The median progression‐free survival (PFS) of the LIPI 0 group and LIPI 1 group was significantly longer than that of the LIPI 2 group (6.6 months vs. 4.0 months, *P* = 0.006 and 5.5 months vs. 4.0 months, *P* = 0.015, respectively) (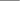 ) LIPI 0 group, (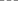 ) LIPI 1 group, (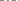 ) LIPI 2 group. The median PFS of the LIPI 0 group was not significantly different from that of the LIPI 1 group (*P* = 0.725). (**b**) The median overall survival (OS) of the LIPI 0 group was significantly longer than that of the LIPI 1 group and LIPI 2 group (17.1 months vs. 11.6 months, *P* = 0.009 and 17.1 months vs. 5.9 months, *P* < 0.001, respectively) (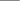 ) LIPI 0 group, (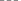 ) LIPI 1 group, (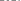 ) LIPI 2 group. The median OS of the LIPI 1 group was significantly longer than that of the LIPI 2 group (*P* = 0.001).

The PFS and OS in ED‐SCLC patients are shown in Figure 3. The PFS of the LIPI 0, 1, and 2 groups was 6.6 months (95% CI: 5.0–8.3 months), 5.5 months (95% CI: 5.0–6.0 months), and 4.0 months (95% CI: 3.7–4.2 months), respectively. The OS of the LIPI 0, 1, and 2 groups was 17.1 months (95% CI: 12.4–21.8 months), 11.6 months (95% CI: 8.4–14.9 months), and 5.9 months (95% CI: 2.8–9.1 months), respectively. The PFS of the LIPI 0 group was significantly longer than that of the LIPI 2 group (*P* = 0.006). The OS of the LIPI 0 group was significantly longer than that of the LIPI 1 group (*P* = 0.009) and LIPI 2 group (*P* < 0.001).

### Patient characteristics and efficacy of treatment in ED‐SCLC patients

The patient characteristics and efficacy of treatment according to LIPI in ED‐SCLC patients are summarized in Table 4. A total of 30 (28.6%), 52 (49.5%), and 23 patients (21.9%) were classified into the LIPI 0, 1 and 2 groups, respectively. In the LIPI 0 group, platinum plus irinotecan was used as a first‐line treatment in 17 patients (56.7%), and 13 patients (43.3%) received platinum plus etoposide. The best objective response to first‐line chemotherapy was as follows: 24 patients (80.0%) had PR, five patients (16.7%) had stable disease (SD), and one patient (3.3%) had PD. The ORR was 80.0% (95% CI: 65.4%–94.6%). The rates of patients in the LIPI 0 group who received second‐ and third‐line chemotherapy were 73.9% and 56.5%, respectively. In the LIPI 1 group, platinum plus irinotecan was used as first‐line treatment in 21 patients (40.4%), 29 patients (56.8%) received platinum plus etoposide, and two patients (3.8%) received palliative care. The best objective response to first‐line chemotherapy was as follows: three patients (5.8%) had CR, 34 patients (65.4%) had PR, seven patients (13.5%) had SD, five patients (9.6%) had PD, and three patients (5.8%) were not evaluated. The ORR was 75.5% (95% CI: 63.3%–87.7%). The rates of patients in the LIPI 1 group who received second‐ and third‐line chemotherapy were 70.0% and 26.3%, respectively. In the LIPI 2 group, platinum plus irinotecan was used as first‐line treatment in seven patients (30.4%), 14 patients (60.9%) received platinum plus etoposide, one patient (4.3%) received oral etoposide, and one patient (4.3%) received palliative care. Regarding the best objective response to first‐line chemotherapy, 13 patients (56.5%) had PR, five patients (21.7%) had SD, three patients (13.0%) had PD, and two patients (8.7%) were not evaluated. The ORR was 61.9% (95% CI: 40.6%–83.2%). The rates of patients in the LIPI 2 group who received second‐ and third‐line chemotherapy were 40.0% and 21.1%, respectively. The rate of third‐line chemotherapy in the LIPI 0 group was significantly higher than that in the LIPI 1 group (*P* = 0.018). The rates of second‐ and third‐line chemotherapy administration in the LIPI 0 group were significantly higher than those in the LIPI 2 group (*P* = 0.025 and *P* = 0.020, respectively). The one‐year survival rate in the LIPI 0 group was significantly higher than that in the LIPI 1 group (75.3% vs. 42.3%, *P* = 0.005) and in the LIPI 2 group (75.3% vs. 4.9%, *P* < 0.001).

**Table 4:** Extensive disease‐small‐cell lung cancer patient characteristics and efficacy of treatment according to lung immune prognostic index

	LIPI, N (%)			
Category	0	1	2	*P*‐value
Patients, N	30	52	23	
Median age (range) years	71 (54–85)	71 (43–86)	72 (59–85)	
Gender, male/female	29 (96.7)/1 (3.3)	43 (82.7)/9 (17.3)	19 (82.6)/4 (17.4)	
ECOG performance status				
0–1/2–3	30 (100.0)/0 (0.0)	37 (71.2)/15 (28.8)	10 (43.5)13 (56.5)	
Stage				
IIIC plus IVA/IVB	18 (60.0)/12 (40.0)	13 (25.0)/39 (75.0)	7 (30.4)/16 (69.6)	
Number of metastatic lesion				
<2/≥2	23 (76.7)/7 (23.3)	24 (46.2)/28 (53.8)	10 (43.5)/13 (56.5)	
First‐line treatment				
Platinum plus irinotecan	17 (56.7)	21 (40.4)	7 (30.4)	
Platinum plus etoposide	13 (43.3)	29 (56.8)	14 (60.9)	
Etoposide (oral)	0 (0.0)	0 (0.0)	1 (4.3)	
Palliative care	0 (0.0)	2 (3.8)	1 (4.3)	
Response to first‐line chemotherapy				
Complete response	0 (0.0)	3 (5.8)	0 (0.0)	
Partial response	24 (80.0)	34 (65.4)	13 (56.5)	
Stable disease	5 (16.7)	7 (13.5)	5 (21.7)	
Progressive disease	1 (3.3)	5 (9.6)	3 (13.0)	
Not evaluated	0 (0.0)	3 (5.8)	2 (8.7)	
Overall response rate, % (95%, CI)	80.0 (65.4–94.6)	75.5 (63.3–87.7)	61.9 (40.6–83.2)	
Rate of second‐line chemotherapy, %	73.9	70.0	40.0	
LIPI 0 vs. LIPI 1				0.741
LIPI 0 vs. LIPI 2				0.025
LIPI 1 vs. LIPI 2				0.025
Rate of third‐line chemotherapy, %	56.5	26.3	21.1	
LIPI 0 vs. LIPI 1				0.018
LIPI 0 vs. LIPI 2				0.020
LIPI 1 vs. LIPI 2				0.754
1‐vear survival rate, %	75.3	42.3	4.9	
LIPI 0 vs. LIPI 1				0.005
LIPI 0 vs. LIPI 2				<0.001
LIPI 1 vs. LIPI 2				0.006

ECOG Eastern Cooperative Oncology Group; LIPI, lung immune prognostic index.

## Discussion

SCLC is sensitive to chemotherapy and radiotherapy; however, the median survival time (MST) has been reported to be 25–34 months for LD‐SCLC and only 10–12 months for ED‐SCLC.^16^, ^17^, ^18^, ^19^ In our institution, the MST of LD‐SCLC and ED‐SCLC were 23.8 months (95% CI: 18.3–29.3 months) and 11.6 months (95% CI: 9.7–13.6 months), respectively. These results were comparable to those of previous reports and have been reflected in clinical practice.^16^, ^17^, ^18^, ^19^

The present study demonstrated for the first time that LIPI was an independent prognostic factor associated with OS, in addition to age, PS, and stage, in SCLC. However, LIPI was not useful as a prognostic factor associated with PFS and OS for LD‐SCLC. Käsmann *et al*.^20^ reported that the OS of the higher NLR (≥ 4) group was significantly longer than that of the lower NLR (< 4) group in LD‐SCLC patients (27 months vs. 10 months, *P* = 0.011). However, Kang *et al*.^21^ reported that the OS of the higher NLR (≥ 4) group was not significantly different from that of the lower NLR (< 4) group in LD‐SCLC patients (17.35 months vs. 12.68 months, *P* = 0.946). The cause of the difference between the two reports may be that LD‐SCLC had a lower systemic inflammatory response than ED‐SCLC and was difficult to be reflected on the NLR. LIPI was correlated with dNLR and LDH; consequently, we should interpret it the same as NLR. Therefore, there was no significant difference in PFS and OS between the LIPI 0 group and the LIPI 1 plus 2 group for LD‐SCLC in the present study.

In the present study, although the analysis results for the LIPI 2 group consisted of only a few cases and were just for reference, according to the examination of five patients in the LIPI 2 group, the OS of the LIPI 2 group was significantly shorter than that of the LIPI 0 and 1 groups (5.0 months vs. 25.5 months, *P* < 0.001 and 5.0 months vs. 18.9 months, *P* = 0.023, respectively). The causes were that two out of five patients had a PS of 3, and the first‐line treatment was chemotherapy alone in all five patients. Therefore, if we accumulate more cases, LIPI 2 may be shown as an unfavorable prognostic factor for LD‐SCLC. The fact that LIPI was not significantly different as a prognostic factor in the multivariate analysis associated with PFS in all SCLC patients was considered to be the consequence of LIPI not being a prognostic factor in LD‐SCLC.

In ED‐SCLC, the PFS of the LIPI 0 and 1 groups was significantly longer than that of the LIPI 2 group. There was a significantly higher proportion of poor PS (2–3) patients in the LIPI 2 group than in the LIPI 0 and 1 groups (56.5% vs. 0.0%, *P* < 0.001 and 56.5% vs. 28.8%, *P* = 0.022, respectively). Previous analysis of 14 SCLC trials suggested that poor PS led to worse PFS in ED‐SCLC.^22^ Therefore, the main reason for shorter PFS in the LIPI 2 group is that patients with poor PS in addition to greater dNLR (≥ 3) and higher LDH (≥ 223 U/L) are significantly included, regardless of the differences in the rate of first‐line chemotherapy and the ORR. However, there was no significant difference between the LIPI 0 and 1 groups, despite the fact that the LIPI 1 group included a greater proportion of poor PS patients than the LIPI 0 group did (*P* = 0.001). Russo *et al*.^13^ reported that in patients who received nivolumab or docetaxel, the PFS of the higher dNLR group was not significantly different from that of the lower dNLR group. In other words, it is suggested that exhibiting both a greater dNLR and higher LDH may be an unfavorable prognostic factor for PFS in ED‐SCLC.

The OS of the LIPI 0 group was significantly longer than that of the LIPI 1 group. Although there was no significant difference in PFS between the two groups, there were two factors that caused a significant difference in OS. First, the rate of stage IVB in the LIPI 1 group was significantly higher than that in the LIPI 0 group (75% vs. 40%, *P* = 0.006, respectively). Shirasawa *et al*.^23^ reported that the OS of ED‐SCLC patients with stage IVA was significantly longer than that of patients with stage IVB (15.2 months vs. 7.3 months, respectively), and Tendler *et al*.^24^ reported that the OS of ED‐SCLC patients with stage IVA was significantly longer than that of patients with stage IVB (8.5 months vs. 5.3 months, respectively). These reports demonstrated that stage IVB is an independent unfavorable prognostic factor for ED‐SCLC. Second, the rate of third‐line treatment in the LIPI 0 group was higher than that in the LIPI 1 group (56.5% vs. 26.3%, *P* = 0.018). The prospective German Tumor Registry Lung Cancer cohort study reported that the median third‐line OS of patients who received third‐line treatment was 5.8 months.^25^ We reported that the median third‐line OS of patients who received third‐line treatment was 5.2 months.^26^ These data suggest that the addition of serial chemotherapies after second‐line therapy could prolong OS in patients with ED‐SCLC. The PFS and OS of the LIPI 0 and 1 groups were significantly longer than those of the LIPI 2 group. These data reflect whether systemic inflammation plays an important role in the inefficacy of chemotherapy in ED‐SCLC.

The present investigation had several limitations. First, it was a retrospective study, with a small number of patients enrolled in the cohort and only included 15 years of patient recruitment. Thus, we could not perform analyses of all patients stratified into LIPI 0, 1, and 2 groups by LIPI score to investigate if it was a prognostic factor. Second, patient treatment in the present study differed greatly.

In conclusion, the present study is the first to demonstrate that pretreatment LIPI is an independent prognostic factor associated with OS for SCLC. LD‐SCLC, including early‐stage disease, may not be useful as a prognostic factor associated with PFS and OS. However, in ED‐SCLC, the OS was significantly different for each LIPI score. In conclusion, LIPI was a more sensitive prognostic factor in ED‐SCLC.

## Disclosure

The authors have no conflicts of interest in this study.
